# Dietary Fermented Chinese Chive Juice Improves Growth Performance and Reshapes the Fresh Meat Volatile Flavor Profile of Small-Tailed Han Sheep

**DOI:** 10.3390/ani16101521

**Published:** 2026-05-15

**Authors:** Ping Sheng, Kaimin Niu, Li He, Chunxia Mao, Shaoshi Ji, Bingbing Huang, Dongsheng Wang, Chunhua Yang

**Affiliations:** Institute of Biological Manufacturing, Jiangxi Academy of Sciences, Nanchang 330096, China

**Keywords:** fermented Chinese chive juice, Small-Tailed Han sheep, phytogenic feed additive, growth performance, fresh meat flavor

## Abstract

This study investigated the effects of fermented Chinese chive (FCC) juice supplementation on growth performance, blood parameters, meat quality, and fresh meat volatile compounds in Small-Tailed Han sheep. Sixty sheep were randomly assigned to a control group (CG; basal diet) or an FCC juice treatment group (TG; basal diet supplemented with 1.0 mL/kg feed offered on an as-fed basis), with three pens per treatment and 10 sheep per pen. FCC juice significantly increased average weight gain (AWG) and average daily gain (ADG) (*p* < 0.05), whereas carcass traits were not significantly affected (*p* > 0.05). Most meat quality traits, antioxidant indices, and serum biochemical parameters were unchanged, although serum glucose (GLU) and cholesterol (CHO) decreased in the FCC juice treatment group (*p* < 0.05). GC × GC-TOF/MS analysis showed that FCC juice altered the volatile profile of fresh sheep meat. Compared with the control group, the FCC juice treatment group showed higher relative contents of aldehydes, ketones, and heterocyclic compounds, but lower contents of hydrocarbons and acids. Seventeen differential volatile compounds were identified using VIP > 1 and FDR-adjusted *p* < 0.05, with esters representing the major upregulated class. Based on the published odor descriptors of the identified volatile compounds, the FCC juice treatment group showed stronger green and apple-like aroma-associated characteristics. Overall, FCC juice showed a modest positive effect on growth performance and altered the volatile compound profile of fresh sheep meat; however, flavor-related findings should be regarded as preliminary because of the limited slaughter sample size and the absence of direct sensory validation.

## Simple Summary

Natural feed additives are receiving increasing attention as alternatives to antibiotic growth promoters in animal production. Chinese chive is a rich source of bioactive compounds, and fermentation may further improve its nutritional and functional properties. However, its application in sheep production has rarely been studied. In this study, fermented Chinese chive juice was supplemented in the diet of Small-Tailed Han sheep to evaluate its effects on growth performance, blood parameters, meat quality, and fresh meat volatile compounds. The results showed that fermented Chinese chive juice modestly improved average weight gain and average daily gain, while having limited effects on most conventional meat quality traits. It also changed some serum metabolic indicators and, importantly, altered the volatile compound profile of fresh sheep meat. Meat from the supplemented group showed increased levels of several aroma-related volatile compounds, particularly those associated with green and apple-like notes. Therefore, these findings indicate that fermented Chinese chive juice may have potential as a natural feed additive in sheep, but its effects on meat flavor require validation using larger slaughter sample sizes and direct sensory evaluation.

## 1. Introduction

Antibiotics have long been incorporated into livestock feed to promote growth performance and support animal health [1,2]. However, the extensive use of antibiotic growth promoters has raised increasing concerns regarding drug residues in animal-derived foods and, more importantly, the emergence and dissemination of antimicrobial resistance, which is now recognized as a major One Health issue. Current regulations governing the use of antibiotic growth promoters across different countries have been comprehensively summarized by Rahman et al. [3]. In line with this global trend, China implemented legislation in 2019 prohibiting the use of certain antibiotic growth promoters, with the exception of traditional Chinese herbal products [4]. Against this background, the development of effective natural alternatives to antibiotic growth promoters has become a major priority in animal nutrition research. Recent studies further indicate that non-antibiotic feed additives, particularly plant-derived preparations, can positively influence animal performance, gut health, and immune responses, supporting their use as multifunctional tools rather than simple growth-promoting substitutes [5].

Among phytogenic resources, plants belonging to the genus *Allium*, including garlic, shallot, onion, chive, and leek, have been widely studied for their beneficial effects on livestock productivity and meat quality [6]. In recent years, Allium-derived additives have increasingly been regarded as promising functional ingredients in antibiotic-free feeding programs because they can simultaneously support animal health and production performance [5].

Chinese chives (CCs), a leafy perennial vegetable of the *Allium* genus, have been traditionally used as food, seasoning, and medicinal material in many global cultures [7,8]. Owing to its perennial growth habit and repeated annual harvesting, large quantities of intermediate biomass and non-marketable aerial parts are generated during cultivation and processing. These by-products are often underutilized despite being rich in protein, vitamins, sulfur-containing compounds, polyphenols, flavonoids, saponins, and fructans [9,10,11]. Therefore, the valorization of CCs by-products as functional feed resources is of increasing interest, not only from a nutritional perspective but also from the standpoint of circular agriculture and sustainable by-product utilization.

Fermentation may further enhance the feeding value of plant materials by improving palatability, releasing or transforming bioactive compounds, reducing anti-nutritional factors, and generating beneficial microbial metabolites. Consistent with this concept, fermented Chinese chive (FCC) preparations exhibit antibacterial, antioxidant, and anti-inflammatory activities, and their dietary inclusion has been shown to improve egg production in laying hens [12], promote early testicular development in suckling piglets [13], and support poultry health by improving blood composition profiles and inhibiting the growth of intestinal pathogens [14]. These findings highlight the promise of FCC preparations as functional feed additives.

Despite the documented bioactivities of Chinese chive and the promising application of fermented Chinese chive preparations in poultry and pigs, evidence regarding their use in sheep remains scarce. In particular, little is known about whether FCC juice can simultaneously influence production performance and the volatile flavor profile of fresh sheep meat. Therefore, the present study investigated the effects of dietary FCC juice supplementation on growth performance, serum biochemical parameters, meat quality, and fresh meat flavor characteristics in Small-Tailed Han sheep, with the aim of providing new evidence for the high-value utilization of fermented Chinese chive as a natural feed additive in ruminant production systems.

## 2. Materials and Methods

### 2.1. Ethics Statement

The protocol was approved by the Institutional Animal Care and Use Committee of the Institute of Biological Manufacturing (JXAS2025-015) on 10 October 2025.

### 2.2. Experimental Design and Treatments

FCC juice was prepared as described previously [15]. CC leaves and tubers were squeezed using a juicer and then filtered through four layers of gauze to obtain the CC juice. To carry out controlled fermentation, the CC juice was added to MRS broth at a concentration of 25% (*v*/*v*), followed by inoculation with Lactobacillus plantarum to a final concentration of approximately 10^7^ CFU/mL, with no adjustment of the initial pH, which was approximately 6.0. Fermentation was carried out at 30 °C for 3 days [16].

Sixty Small-Tailed Han sheep (approximately 2 months old, initial body weights (IBWs) 35 ± 2.0 kg) were stratified by initial body weight and then randomly assigned to two dietary treatments, with three replicate pens per treatment and 10 sheep per pen. The dietary treatments consisted of a basal diet without FCC juice supplementation (control group, CG) or the basal diet supplemented with FCC juice at 1.0 mL/kg feed offered on an as-fed basis (treatment group, TG) (Table 1). Because the FCC juice was an aqueous preparation added at a very low inclusion level, its contribution to dietary dry matter and energy was considered negligible and was not included in the calculated nutrient composition. Both groups received the same basal diet, which was formulated to meet the nutrient requirements recommended by NRC (2007). The experiment included a 12-day adaptation period to allow animals to acclimate to the diet, pen environment, and FCC juice administration, followed by a 60-day feeding trial. The initial body weights (IBWs) were determined before the start of the feeding trial. Feed was offered twice daily at 07:00 and 18:00, with the sheep having unrestricted access to both feed and water.

### 2.3. Sampling and Measurement

On a daily basis, feed refusals were collected, followed by weighing to calculate average daily feed intake (ADFI) based on feed offered and residual feed. Individual body weights were recorded on days 1 and 60 of the trial to assess IBW and final body weight (FBW). Average weight gain (AWG) and average daily gain (ADG) were estimated by subtracting IBW from FBW. The feed-to-gain ratio (F/G) was calculated as the ratio of ADFI to ADG. ADFI, IBW, FBW, AWG, ADG and F/G were calculated using pen as the experimental unit.

On completion of the feeding trial, the animals were fasted overnight while water remained available. Ten sheep (five per group) were then selected for slaughter. Prior to slaughter, blood samples from the jugular vein were collected into 5 mL serum separator tubes, followed by centrifugation (3000× *g*, 15 min, 4 °C). For further analysis, the supernatant was separated. Following slaughter, the heads of the animals were detached at the atlanto-occipital joint and weighed, while the feet were removed at the respective carpal and tarsal joints and weighed. The loin-eye area (cm^2^) was measured on the cross-section of the longissimus dorsi muscle between ribs 12 and 13. Longissimus dorsi samples were harvested within 20 min post-mortem, visible fat was removed, and the tissues were kept at 4 °C until analysis.

Blood biochemical indices, including total superoxide dismutase (T-SOD), glutathione peroxidase (GPx), catalase (CAT), reduced glutathione (GSH), malondialdehyde (MDA), lysozyme (LZM), alkaline phosphatase (ALP), total protein (TP), albumin (ALB), urea (UREA), triglycerides (TG), total cholesterol (CHO), high-/low-density lipoprotein (HDL/LDL), and non-esterified fatty acids (NEFA) were assessed using an automatic analyzer (IDEXX Catalyst One Chemistry Analyzer, IDEXX Laboratories, Inc., Westbrook, ME, USA) according to the manufacturer’s instructions.

Meat samples were analyzed for moisture, crude protein, crude fat, and ash content according to AOAC methods [17]. Cooking loss was determined by weighing samples before and after cooking in a water bath at 80 °C for 1 h, following the method described by Babiker et al. [18]. Cooking loss represented weight loss as a percentage of the initial sample weight. The loin-eye area was calculated using the formula: muscle width × muscle height × 0.7.

For hydrolyzed amino acid determination, samples were accurately weighed into glass bottles, followed by the addition of 6 mol/L HCl. After flushing with nitrogen to remove oxygen, the samples underwent hydrolysis for 22–24 h at 110 °C, after which the solutions were transferred to 50 mL volumetric flasks, followed by adding ultrapure water to bring them up to volume. Hydrolyzed amino acid profiles were quantified using a Hitachi LA-8080 analyzer (Hitachi High-Tech Corporation, Minato City, Tokyo, Japan). Total lipids were extracted for fatty acid analysis as described by Folch et al. using a QP 2010 Ultra GC-MS instrument (Shimadzu Corporation, Kyoto City, Japan) with a fused silica capillary column (100 m × 0.25 mm internal diameter × 0.2 μm film thickness) with helium as carrier gas [19]. The temperature program was 5 min at 100 °C, rising to 240 °C at a rate of 4 °C/min, and maintenance at 240 °C for 30 min. Individual fatty acids were identified by comparing their retention times with those of authentic fatty acid methyl ester (FAME) standards analyzed under identical conditions. Fatty acid levels were evaluated as percentages of total fatty acids identified in this study.

A LECO Pegasus^®^ 4D system (LECO Corporation, St. Joseph, MI, USA; ChromaTOF software v4.22) was utilized to assess volatile flavor compounds, consisting of an Agilent 8890A gas chromatograph (Agilent Technologies, Inc., Santa Clara, CA, USA) equipped with a split/splitless injector, a dual-stage cryogenic modulator (LECO Corporation, St. Joseph, MI, USA), and a time-of-flight mass spectrometer (TOF-MS; LECO Corporation, St. Joseph, MI, USA). A DB-Heavy Wax column (30 m × 250 μm I.D., 0.5 μm film thickness; Agilent Technologies, Inc., USA) represented the first-dimension (1D) column, while an Rxi-5Sil MS column (2.0 m × 150 μm I.D., 0.15 μm film thickness; Restek Corporation, Bellefonte, PA, USA) served as the 2D column. The carrier gas was high-purity helium (>99.999%) with a flow rate of 1.0 mL/min. The primary oven temperature was 40 °C for 3 min, raised to 250 °C at 5 °C/min and maintained for 5 min. The secondary oven temperature was 5 °C higher than that of the primary, while the temperature of the modulator was 15 °C higher than the temperature of the secondary column. A 4.0 s modulation period was utilized with an injector temperature of 250 °C.

Flavor compound detection was performed using the Pegasus BT 4D system (LECO Corporation, St. Joseph, MI, USA; ChromaTOF software v5.32). The temperatures of both the transfer line and TOF-MS ion source were 250 °C. Data were acquired at a rate of 200 spectra/s. The electron ionization (EI) mode was 70 eV, scanning a mass range of m/z 35–550 with a detector voltage of 1960 V. Volatile compounds were putatively annotated using mass spectral matching against commercial mass spectral libraries and chromatographic information, including retention index information when available. Because authentic standards were not used for all detected volatile compounds, the total number of detected compounds should be interpreted as putative annotations rather than fully validated identifications.

Odor descriptors were assigned based on reported odor characteristics of identified volatile compounds from published databases and literature. No trained sensory panel evaluation was conducted in the present study. Therefore, descriptors such as green, fruity, apple-like, buttery, or sweet refer to compound-associated odor annotations rather than direct sensory scores.

### 2.4. Statistical Analysis

Data were analyzed using SPSS 22.0 (IBM Corp., Armonk, NY, USA). For pen-level traits, including ADFI, IBW, FBW, AWG, ADG and F/G, pen was used as the experimental unit. For individual-level traits, pen was included as a random effect where appropriate to account for the housing design. Results are presented as mean ± standard error (SE). Statistical significance was declared at *p* < 0.05. For volatile compound comparisons, *p* values were adjusted for multiple testing using the Benjamini–Hochberg false discovery rate (FDR) procedure. Compounds with VIP >1 and FDR-adjusted *p* < 0.05 were considered differential volatile compounds. Partial least squares-discriminant analysis (PLS-DA) was conducted as a supervised classification model to evaluate differences in volatile compound profiles among treatment groups. The performance and reliability of the PLS-DA model were assessed using R^2^X, R^2^Y, and cross-validated Q^2^ values, and permutation testing was further performed to evaluate the risk of model overfitting.

## 3. Results

### 3.1. Growth Performance and Carcass Traits

Growth performance and slaughter characteristics, including IBW, FBW, AWG, ADG, ADFI, F/G, dressing percentage, cooking loss percentage, and loin-eye area, are summarized in Table 2. There were no significant differences in IBW and FBW between the CG and TG groups (*p* > 0.05). Nevertheless, FCC juice supplementation significantly increased AWG and ADG compared with the control (*p* < 0.05). The AWG of sheep in the TG group was 17.67 ± 0.58 kg, which was significantly higher than that in the CG group (15.68 ± 0.87 kg). Likewise, ADG was significantly elevated in the TG group (0.29 ± 0.01 kg) relative to the CG group (0.26 ± 0.00 kg). The F/G ratio was significantly lower in the TG group than in the CG group (3.06 ± 0.07 vs. 3.46 ± 0.09, *p* < 0.05), suggesting improved feed efficiency after FCC juice supplementation.

For carcass traits, no significant differences were detected in dressing percentage, cooking loss percentage, or loin-eye area between the two groups (*p* > 0.05). Although FCC juice supplementation slightly increased dressing percentage and decreased cooking loss percentage, these changes did not reach statistical significance (Table 2).

### 3.2. Meat Quality

The quality parameters of the longissimus dorsi muscles from the CG and TG groups are presented in Table 3. There were no inter-group differences in moisture, crude fat, or crude ash contents (*p* > 0.05), whereas the TG group exhibited markedly reduced crude protein content relative to the controls (*p* < 0.05).

Table 4 showed the effect of FCC juice on the amino acid composition of the longissimus dorsi muscle in Small-Tailed Han sheep. Compared with the CG group, the TG group exhibited significantly lower contents of Met and Pro (*p* < 0.05). No significant differences were detected for the remaining amino acids (*p* > 0.05).

As shown in Table 5, FCC juice supplementation had no significant effect on the fatty acid composition of the longissimus dorsi muscle in Small-Tailed Han sheep. No significant differences were observed between the CG and TG groups in the proportions of saturated fatty acids, monounsaturated fatty acids, or polyunsaturated fatty acids (*p* > 0.05). Specifically, C14:0, C16:0, C18:0, C14:1, C16:1, C18:1 n9c, C20:1, C18:2n6c, C18:3n3, C20:3n6, C20:3n3, C20:4n6, C20:5n3, and C22:6n3 were all unaffected by treatment. These findings suggest that FCC juice did not markedly modify the fatty acid profile of the longissimus dorsi muscle.

### 3.3. Blood Parameters

**Figure 1:**
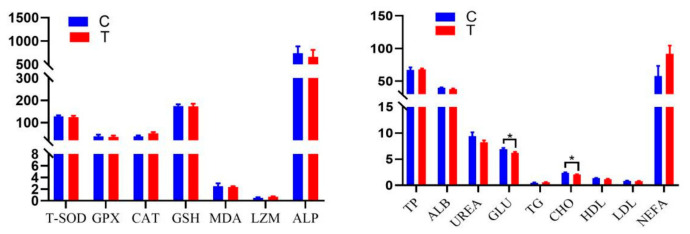
Effect of dietary FCC juice supplementation on blood characteristics in Small-Tailed Han sheep. “*” indicates significant difference at *p* < 0.05.

The comparison between the CG and TG groups revealed that most antioxidant indices, including T-SOD, GPx, CAT, GSH, MDA, LZM, and ALP were not markedly affected by the treatment. Regarding serum biochemical parameters, TP, ALB, UREA, TG, HDL, and LDL showed no substantial variation between groups. By contrast, GLU and CHO levels were reduced in the TG group (*p* < 0.05), while NEFA content was increased relative to the CG group (Figure 1). These findings suggest that the treatment exerted a limited influence on antioxidant capacity overall but significantly modulated several metabolic-related biochemical indicators.

### 3.4. Characterization of the Volatile Flavor Profiles

**Figure 2:**
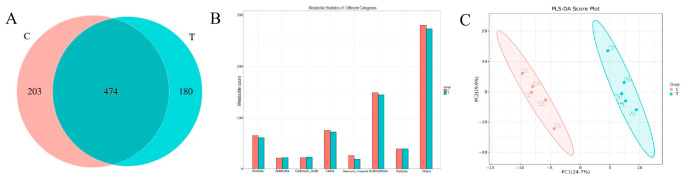
Analysis of the volatile flavor characteristics of CG and TG group. (**A**) Venn diagram of the identified volatile compounds in two groups. (**B**) Classification of identified volatile compounds. (**C**) PLS–DA analysis of the identified flavor compounds from all samples.

To characterize volatile flavor compounds in Small-Tailed Han sheep meat, GC × GC-TOF/MS analysis was conducted for both CG and TG samples. Overall, 677 and 654 volatile features were putatively annotated in the CG and TG samples, respectively, with 474 features shared between the two groups (Figure 2A). These results indicated a substantial overlap in the annotated volatile profile between groups, together with treatment-associated differences. Because most compounds were annotated using library matching rather than authentic standards, the numbers should be interpreted as putative annotations.

Classification of these compounds revealed that they were primarily composed of hydrocarbons (5.94–6.49%), alcohols (14.64–15.41%), aldehydes (6.93–7.64%), ketones (10.08–12.11%), esters (10.48–12.78%), acids (2.69–4.11%), and heterocyclic compounds (0.76–1.04%) in both groups (Figure 2B). Relative to the controls, the TG group exhibited higher relative contents of aldehydes, ketones and heterocyclic, whereas hydrocarbons and acids were present at lower levels (Figure 2B). The PLS-DA model showed R^2^X = 0.446, R^2^Y = 0.994, and Q^2^ = 0.901, indicating good discrimination and strong predictive ability among the treatment groups. The high R^2^Y value suggested good model fitting, while the high cross-validated Q^2^ value reflected strong predictive robustness. In addition, permutation testing showed that the Q^2^ values of the permuted models were lower than that of the original model, with a Q^2^ intercept of 0.10, suggesting that the model was not mainly driven by random classification. These results supported the reliability of the PLS–DA model and confirmed that FCC treatment induced distinct changes in the volatile compound profile of fresh sheep meat (Figure 2C).

### 3.5. Identification of Differential Flavor Compounds

Using variable importance in projection (VIP) values > 1 and FDR-adjusted *p* < 0.05 as selection criteria, 17 volatile compounds with differential abundance were identified between the CG and TG groups, including 10 upregulated and 7 downregulated compounds (Table 6). The upregulated compounds mainly included esters, organoheterocyclic compounds, ketones, hydrocarbons, and organic acids and derivatives, with esters representing the predominant class. The downregulated compounds were mainly distributed among benzenoids, esters, and ketones. In particular, the increase in several ester compounds suggests that ester-related volatile formation may contribute to FCC treatment-associated changes in meat odor descriptors.

### 3.6. Analysis of Flavor Compounds

**Figure 3:**
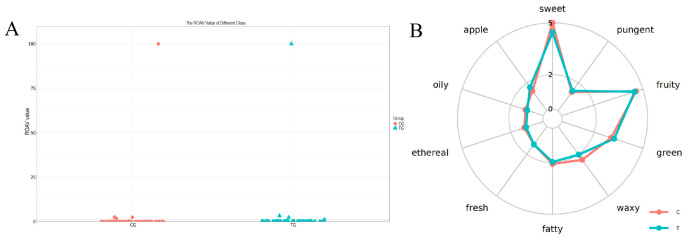
Analysis of aroma-active compounds and compound-associated odor descriptors in CG and TG groups. (**A**) ROAV analysis of the identified volatile compounds of CG and TG groups. (**B**) Odor descriptors assigned according to reported odor characteristics of identified volatile compounds.

Aroma-active compounds in the meat of the two treatment groups were identified using the relative odor activity value (ROAV) approach. The results indicated that a total of four aroma compounds exhibited ROAVs greater than 1 in at least one of the meat samples (Figure 3A; Table 7), suggesting that only a small subset of the putatively annotated volatiles were predicted to make major contributions to overall aroma based on concentration and odor threshold. Among them, 2,3-butanedione displayed the highest ROAVs in both the CG and TG groups. The remaining three aroma-active compounds (2-nonenal, heptanal, and 2-pentylfuran) were also detected in both groups. The TG group exhibited a higher contribution of volatile compounds associated with green and apple-like notes, indicating a possible enhancement of fresh and fruity aroma characteristics. However, these descriptors were assigned based on reported odor characteristics of the identified volatile compounds in databases and the literature, and no trained sensory panel was conducted in this study. Therefore, the green and apple-like notes in the TG group should be regarded as inferred aroma attributes rather than direct sensory evaluation results, and they require further validation by trained panel assessment (Figure 3B).

In addition, sensory characterization of the overall flavor profiles revealed clear differences between treatments. Meat from the TG group exhibited more pronounced green and apple-like odor descriptor profile compared with meat from the CG group (Figure 3B).

## 4. Discussion

The present study demonstrates that dietary fermented Chinese chive juice may act as a phytogenic feed additive with modest growth-related effects and the capacity to alter the volatile compound profile of fresh sheep meat. Under the present experimental conditions, FCC juice improved growth performance, selectively altered serum metabolic indicators, and markedly reshaped the volatile composition and compound-associated odor descriptors of fresh meat.

### 4.1. Effects of FCC Juice on Growth Performance and Meat Quality

Growth performance is a key determinant of economic efficiency in livestock production and is commonly evaluated using body weight, average weight gain, feed intake, and feed conversion efficiency. In the present study, FCC juice supplementation significantly increased AWG and ADG, whereas IBW and FBW did not differ significantly between the two groups. In addition, ADFI was almost unchanged, while the F/G ratio was significantly reduced in the TG group. These results suggest that FCC juice did not promote growth by increasing feed intake but may have improved feed utilization efficiency. The lower F/G ratio further indicates that sheep receiving FCC juice required less feed to achieve comparable or greater weight gain, which may have practical relevance for improving production efficiency.

The growth-promoting effect of FCC juice may be partly associated with bioactive compounds present in Chinese chive, including organosulfur compounds, flavonoids, and other phenolic constituents [20]. This interpretation is indirectly supported by previous ruminant studies showing that *Allium mongolicum* Regel, essential oil or water extract can improve ADG, nutrient digestibility, rumen fermentation characteristics, and bacterial communities in sheep or calves [21,22]. Comparable growth-promoting effects have also been reported for other *Allium* plants and their derivatives, such as onion bulbs, garlic powder, and Chinese chives [14,23,24]. Therefore, the increased AWG and ADG, together with the reduced F/G ratio observed in the present study, may be related to improved nutrient utilization rather than increased feed consumption. However, because rumen fermentation parameters and nutrient digestibility were not directly measured in this study, this explanation should be regarded as a plausible hypothesis and requires further verification.

In contrast to the positive effects on growth performance, FCC juice supplementation decreased the crude protein content of the longissimus dorsi muscle, while moisture, crude fat, and ash contents were not significantly affected. Meanwhile, Met and Pro contents were significantly reduced, and most other amino acids showed numerically lower values in the TG group. These results indicate that FCC juice did not improve the protein-related nutritional quality of meat and may have had a potential adverse effect on muscle protein deposition or certain amino acid fractions. Therefore, the effect of FCC juice on meat quality should be interpreted cautiously rather than as uniformly beneficial.

The decreases in Met and Pro may partly reflect the overall reduction in muscle crude protein content. Met is an essential sulfur-containing amino acid involved in protein synthesis and related metabolic processes, whereas Pro is closely associated with collagen- and connective tissue-related protein fractions. However, because plasma amino acid profiles, nitrogen balance, collagen content, hydroxyproline concentration, and muscle protein turnover were not measured, the biological significance and underlying cause of the reduced Met and Pro contents cannot be directly determined.

One possible explanation for these protein- and amino acid-related changes may be altered nutrient utilization, absorbable amino acid supply, or nutrient partitioning. Previous ruminant studies have reported that *Allium mongolicum* Regel-derived preparations can affect rumen fermentation, nutrient digestibility, microbial protein production, and nitrogen utilization [21,22]. These findings suggest that *Allium*-derived bioactive compounds may influence nutrient utilization in ruminants. Nevertheless, FCC juice differs from *Allium mongolicum* Regel preparations, and rumen fermentation parameters, nutrient digestibility, nitrogen metabolism, and muscle protein turnover were not assessed in the present study. Thus, the mechanism underlying the reduced crude protein, Met, and Pro contents remains speculative and requires further investigation.

The fermentation process itself may also have contributed to the biological efficacy of FCC juice. Fermentation can increase the bioavailability of phytochemicals, generate small bioactive metabolites, improve palatability, and enhance the functional value of plant materials. A recent study reported that fermented chive with *Lactobacillus plantarum* improved growth performance, gut health, antioxidant activity, and immune responses in broilers challenged with *Escherichia coli*, supporting that fermentation can potentiate the health-promoting properties of chive-based additives [25]. In the present study, the unchanged ADFI suggests that FCC juice did not negatively affect diet acceptability, while the improved ADG and F/G indicate a potential enhancement of growth efficiency.

### 4.2. Effects of FCC Juice on Blood Biochemical Parameters

Blood biochemical indices provide important objective indicators of animal health status [26]. In the present study, FCC juice supplementation significantly decreased serum CHO and GLU concentrations but increased NEFA concentration. The reduction in GLU may reflect altered glucose supply or utilization, whereas the increase in NEFA may indicate enhanced fatty acid mobilization as an alternative energy substrate. These results suggest that FCC juice may influence energy and lipid metabolism in sheep. Previous studies have reported reduced serum CHO levels in broilers supplemented with FCC, garlic powder, or onion powder [14,27,28]. In addition, allicin-related study in rat model has demonstrated that Allium-derived organosulfur compounds can reduce blood glucose and serum lipid levels and improve metabolic regulation [29]. These findings provide indirect evidence that bioactive compounds in Chinese chive may participate in metabolic regulation. However, because these studies were conducted mainly in non-ruminant species, the relevance of these findings to sheep should be interpreted cautiously.

### 4.3. Effects of FCC Juice on the Volatile Flavor Profile of Fresh Meat

Meat flavor, a key factor in consumer acceptance, is derived from a complex mix of volatile compounds [30]. In the present study, hydrocarbons, alcohols, ketones, esters, acids, and heterocyclic compounds were found to predominate in both treatment groups. This volatile profile is consistent with previous reports on sheep meat flavor composition [31,32].

A major finding of this work is that FCC juice modified the volatile flavor profile of the longissimus dorsi muscle. Although the two groups shared a large number of volatile compounds, PLS-DA clearly separated CG and TG group, demonstrating that FCC juice altered the overall aroma matrix rather than a few isolated compounds. Among the upregulated compounds, the enrichment of multiple esters is particularly noteworthy. Esters are widely recognized as important contributors to fruity, floral, sweet, and fresh odor attributes in food systems, and although they are more often emphasized in fruits and fermented products, they can also increase aroma pleasantness in meat when present at appropriate levels. Reviews on aroma chemistry have consistently noted that esters are closely associated with fruity and floral sensory impressions, and recent sensory work has further shown that esters can enhance sweet, fruity, and floral aroma perception through additive or synergistic effects [33]. Therefore, the increases in ethyl tiglate, propanoic acid ethyl ester, and butanoic acid ethyl ester suggest that fermented Chinese chive juice may have enhanced pleasant fresh aroma characteristics in the meat. This interpretation is consistent with the increased abundance of volatile compounds associated with green and apple-like notes in the TG group. In particular, ethyl tiglate has been identified as a natural volatile constituent of apple and is associated with fruity aroma characteristics, supporting its possible contribution to the apple-like note detected here [34]. By contrast, the downregulation of p-cresol and related phenolic compounds may have reduced characteristic sheepy or phenolic odor intensity. These descriptors were based on published odor characteristics of identified volatile compounds, not on a trained sensory panel. Thus, the effects of FCC juice on perceived meat flavor still require further validation through direct sensory evaluation.

The increased abundance of dihydro-4-methyl-2(3H)-furanone, dihydro-5-methyl-2(3H)-furanone, and 2-octanone may also have contributed to changes in compound-associated odor descriptors in the TG group. Furanone derivatives are widely regarded as potent aroma-active compounds due to their low odor thresholds and their characteristic sweet, caramel-like sensory attributes [35]. Previous studies have described hydroxyfuranones, especially furaneol-related compounds, as key contributors to sweet/caramel-like aroma in a wide range of foods, with perceptible effects even when present at low concentrations [36]. Meanwhile, 2-octanone, as a medium-chain methyl ketone, has been associated with fruity, creamy, fatty, or cheesy nuances, depending on the food matrix and concentration. Recent aroma studies have emphasized that methyl ketones such as 2-octanone can make meaningful contributions to aroma complexity [37]. Fresh meat aroma is determined by the interaction of numerous low- and medium-abundance volatiles derived from lipid oxidation and related precursor pathways, the simultaneous increase in esters, ketones, and selected heterocyclic compounds in the TG group likely contributed to the clear discrimination observed in the multivariate analysis [38], but the sensory significance of these changes remains to be verified experimentally.

### 4.4. Key Aroma-Active Compounds Identified by ROAV Analysis

The ROAV method is widely used to identify key aroma-active compounds in complex flavor systems [39]. Compounds with higher ROAVs contribute more substantially to overall flavor perception [40]. In this study, only four compounds showed ROAV values greater than 1, namely 2,3-butanedione, heptanal, 2-nonenal, and 2-pentylfuran. The small number of ROAV-active compounds is not inconsistent with the large number of putatively an-notated volatiles, because ROAV depends on both relative abundance and odor threshold, many compounds may be present but below their threshold contribution. 2,3-butanedione exhibited the highest ROAV value (100) in both treatment groups, followed by heptanal (2.44–3.31), 2-nonenal (2.37–2.56), and furan (1.13–1.68). These results align with previous reports on meat aroma composition [39,41,42]. Notably, 2,3-butanedione is known to exert a strong influence on meat flavor, imparting caramel-like and sweet sensory attributes. Earlier studies have shown that 2,3-butanedione contributes a buttery aroma to beef and that its concentration is positively associated with overall flavor intensity and consumer acceptance [43]. However, ROAV analysis is an instrumental estimation based on volatile concentration and odor threshold values and therefore cannot fully replace human sensory evaluation. Future research should integrate GC × GC-TOF/MS analysis, ROAV evaluation, and trained sensory panel assessments to further verify whether the detected alterations in aroma compounds can be effectively perceived by consumers.

### 4.5. Limitations

Several limitations should be considered when interpreting the present findings. First, only five animals per treatment were slaughtered, limiting statistical power for carcass traits, meat quality, amino acid composition, fatty acid composition, and volatile compound profiling analysis. Second, the FCC juice was not fully characterized for all potentially active phytochemical and sulfur-containing compounds in the present batch. Third, the study used a single FCC dose, so the optimal supplementation level remains unknown. Finally, odor descriptors were inferred from volatile compound information and were not obtained from a trained sensory panel. These limitations should be addressed in future studies.

## 5. Conclusions

Fermented Chinese chive juice supplementation showed a modest positive effect on growth performance in Small-Tailed Han sheep by increasing average weight gain and average daily gain, while exerting limited effects on carcass traits, muscle fatty acid composition, and most conventional meat quality parameters. It also selectively modulated serum metabolic indicators, as reflected by decreased glucose and cholesterol levels. More importantly, FCC juice altered the volatile compound profile of fresh sheep meat, particularly by changing the abundance of esters and other aroma-related compounds associated with green and apple-like notes. However, because only five animals per treatment were slaughtered and no trained sensory panel was conducted, the flavor-related results should be considered preliminary. Overall, FCC juice may have potential as a natural phytogenic feed additive in sheep, but further studies with larger sample sizes, full additive characterization, and direct sensory validation are required.

## Figures and Tables

**Table 1:** Dietary ingredients and chemical composition of basal diet (DM basis, %).

Diet Composition		Chemical Composition	
Ingredients	Content %	Items	Content
Corn	66.6	Crude protein, %DM	12.14
Soybean meal	10.3	Neutral detergent fiber, %DM	21.58
Peanut vine	10.2	Ether extract, %DM	3.01
Hay	7.7	Calculated metabolizable energy, MJ/kg DM	11.56
4% Premix	3.6	Total phosphorus, %DM	0.35
NaHCO_3_	1.6	Calcium, %DM	0.67

**Table 2:** Effect of FCC juice on growth performance and carcass traits in Small-Tailed Han Sheep.

Item	CG	TG	*p* Value
Growth performance			
IBW, kg	35.31 ± 0.46	34.74 ± 0.21	0.33
FBW, kg	50.99 ± 0.42	52.41 ± 0.61	0.13
AWG, kg	15.68 ± 0.87	17.67 ± 0.58	0.03
ADG, kg	0.26 ± 0.003	0.29 ± 0.01	0.02
ADG, kg per pen	2.61 ± 0.02	2.94 ± 0.08	0.02
ADFI, kg per pen	9.02 ± 0.20	9.01 ± 0.51	>0.99
F/G	3.46 ± 0.09	3.06 ± 0.07	0.01
Carcass traits			
Dressing percentage, %	52.64 ± 0.99	53.61 ± 0.56	0.45
Cook loss percentage, %	33.71 ± 1.56	32.22 ± 2.79	0.70
Loin-eye area, cm^2^	21.96 ± 1.46	21.93 ± 1.03	0.99

IBW: initial body weight; FBW: final body weight; AWG: average weight gain; ADG: average daily gain; ADFI: average daily feed intake; F/G: feed-to-gain ratio. Individual body weights were recorded, but growth performance data were summarized and analyzed using pen as the experiment unit (*n* = 3 pens per treatment). Carcass traits were measured in slaughtered animals (*n* = 5 per treatment).

**Table 3:** Effect of FCC juice on chemical-physical properties of Small-Tailed Han Sheep meat.

Item (%)	CG	TG	*p* Value
Moisture	71.39 ± 0.17	71.86 ± 0.58	0.51
Crude protein	22.58 ± 0.35	21.50 ± 0.04	0.03
Crude fat	3.28 ± 0.33	3.66 ± 0.53	0.60
Ash	0.94 ± 0.09	1.05 ± 0.17	0.64

**Table 4:** Effect of FCC juice on amino acid composition in the longissimus dorsi muscle of Small-Tailed Han Sheep.

Item (%)	CG	TG	*p* Value
Asp	6.62 ± 0.13	6.21 ± 0.14	0.09
Thr	3.26 ± 0.07	3.06 ± 0.07	0.10
Ser	2.7 ± 0.05	2.56 ± 0.06	0.09
Glu	11.14 ± 0.21	10.40 ± 0.22	0.07
Gly	3.17 ± 0.06	2.95 ± 0.06	0.07
Ala	4.51 ± 0.09	4.28 ± 0.09	0.17
Val	3.73 ± 0.08	3.58 ± 0.08	0.30
Cys	0.52 ± 0.02	0.54 ± 0.02	0.44
Met	1.97 ± 0.04	1.82 ± 0.04	0.03
Ile	3.51 ± 0.07	3.29 ± 0.07	0.13
Leu	6.14 ± 0.12	5.79 ± 0.12	0.11
Tyr	2.51 ± 0.05	2.36 ± 0.05	0.08
Phe	2.96 ± 0.06	2.80 ± 0.05	0.12
Lys	6.35 ± 0.12	5.93 ± 0.14	0.09
His	2.09 ± 0.03	1.95 ± 0.05	0.07
Arg	4.51 ± 0.09	4.22 ± 0.09	0.10
Pro	2.83 ± 0.06	2.56 ± 0.05	0.01

**Table 5:** Effect of FCC juice on fatty acid composition in the longissimus dorsi muscle of Small- Tailed Han Sheep (% of total fatty acids).

Item (%)	CG	TG	*p* Value
SFAS			
C14:0	2.23 ± 0.21	2.11 ± 0.16	0.70
C16:0	23.82 ± 0.74	24.07 ± 0.38	0.80
C18:0	13.68 ± 0.85	12.16 ± 0.32	0.17
MUFAS			
C14:1	0.17 ± 0.02	0.17 ± 0.01	0.94
C16:1	2.21 ± 0.21	2.32 ± 0.15	0.72
C18:1, n9c	45.12 ± 0.53	46.84 ± 1.01	0.21
C20:1	0.11 ± 0.01	0.10 ± 0.00	0.46
UFAS			
C18:2n6c	7.11 ± 1.04	7.00 ± 1.03	0.95
C18:3n3	0.50 ± 0.04	0.45 ± 0.03	0.36
PUFAS			
C20:3n6	0.19 ± 0.02	0.18 ± 0.02	0.75
C20:3n3	0.04 ± 0.00	0.04 ± 0.00	0.74
C20:4n6	1.77 ± 0.19	1.68 ± 0.24	0.82
C20:5n3	0.19 ± 0.02	0.16 ± 0.02	0.49
C22:6n3	0.10 ± 0.01	0.10 ± 0.01	0.84

C14:0, myristic acid; C16:0, palmitic acid; C18:0, stearic acid; C14:1, myristoleic acid; C16:1, palmitoleic acid; C18:1 n9c, oleic acid; C18:2 n6c, linoleic acid; C18:3 n3, α-linolenic acid; C20:3 n6, cis-8, 11,14-eicosatrienoic acid; C20:3 n3, cis-11,14,17-eicosatrienoic acid; C20:4 n6, arachidonic acid; C20:5 n3, cis-5,8,11,14,17-eicosapentaenoic acid; C22:6 n3, cis-4,7,10,13,16,19-docosahexaenoic acid.

**Table 6:** Types of differential flavor compounds between CG and TG groups.

Name	Class	FC	FDR	VIP	Regulation
2(3H)-Furanone, dihydro-4-methyl-	Organoheterocyclic compounds	2.63	0.00	2.07	Up
Ethyl tiglate	Esters	2.63	0.00	2.03	Up
2-Octanone	Ketones	1.66	0.00	1.96	Up
Butanoic acid, 3-hydroxy-, ethyl ester	Organic acids and derivatives	2.08	0.01	1.94	Up
2(3H)-Furanone, dihydro-5-methyl-	Organoheterocyclic compounds	1.4	0.01	1.91	Up
Cyclopentane, pentyl-	Hydrocarbons	1.56	0.01	1.88	Up
Propanoic acid, ethyl ester	Esters	1.45	0.01	1.88	Up
Butanoic acid, ethyl ester	Esters	1.98	0.02	1.84	Up
Hexanoic acid, 2-ethyl-, ethyl ester	Esters	1.59	0.03	1.81	Up
Butanedioic acid, diethyl ester	Esters	1.58	0.04	1.78	Up
p-Cresol	Benzenoids	0.17	0.00	2.06	Down
Benzeneacetic acid, ethyl ester	Benzenoids	0.3	0.00	2.03	Down
Butanoic acid, 2-methyl-, ethyl ester	Esters	0.5	0.00	2.00	Down
Phenol, 2-methyl-	Benzenoids	0.65	0.00	1.94	Down
Pentanoic acid, 4-methyl-, ethyl ester	Esters	0.4	0.00	1.92	Down
Propanoic acid, 2-methyl-, 3-hydroxy-2,2,4-trimethylpentyl ester	Esters	0.5	0.03	1.80	Down
5-Hepten-2-one, 6-methyl-	Ketones	0.57	0.04	1.77	Down

**Table 7:** The aroma active compounds with ROAV > 1 in CG and TG groups.

Name	Formula	TG_ROAV	CG_ROAV
2,3-Butanedione	C4H6O2	100	100
2-Nonenal, (E)-	C9H16O	2.37	2.56
Heptanal	C7H14O	3.31	2.44
Furan, 2-pentyl-	C9H14O	1.13	1.68

## Data Availability

The original data generated for this study are included in the article. Further inquiries can be directed to the corresponding author.

## Abbreviations

The following abbreviations are used in this manuscript:

FCC	Fermented Chinese Chive
CC	Chinese Chive
CG	Control Group
TG	Treatment Group
IBWs	Initial Body Weight
ADFI	Average daily feed intake
FBW	Final Body Weight
AWG	Average Weight Gain
ADG	Average Daily Gain
F/G	Feed-to-gain ratio
T-SOD	Total Superoxide Dismutase
GPx	Glutathione Peroxidase
CAT	Catalase
GSH	Reduced Glutathione
MDA	Malondialdehyde
LZM	Lysozyme
ALP	Alkaline Phosphatase
TP	Total Protein
ALB	Albumin
UREA	Urea
TRIG	Triglycerides
CHO	Total Cholesterol
HDL/LDL	High-/Low-Density Lipoprotein
NEFA	Non-Esterified Fatty Acids
Asp	Aspartic acid
Thr	Threonine
Ser	Serine
Glu	Glutamic acid
Gly	Glycine
Ala	Alanine
Val	Valine
Cys	Cysteine
Met	Methionine
Ile	Isoleucine
Leu	Leucine
Tyr	Tyrosine
Phe	Phenylalanine
Lys	Lysine
His	Histidine
Arg	Arginine
Pro	Proline
ROAV	Relative Odor Activity Value

## References

[1] Bedford M. (2000). Removal of antibiotic growth promoters from poultry diets: Implications and strategies to minimize subsequent problems. Worlds Poult. Sci. J..

[2] Cheng G.Y., Hao H.H., Xie S.Y., Wang X., Dai M.H., Huang L.L., Yuan Z.H. (2014). Antibiotic alternatives: The substitution of antibiotics in animal husbandry?. Front. Microbiol..

[3] Rahman M.R.T., Fliss I., Biron E. (2022). Insights in the development and uses of alternatives to antibiotic growth promoters in poultry and swine production. Antibiotics.

[4] Wen R., Li C., Zhao M., Wang H., Tang Y. (2022). Withdrawal of antibiotic growth promoters in China and its impact on the foodborne pathogen *Campylobacter coli* of swine origin. Front. Microbiol..

[5] Wang J., Deng L.F., Chen M.X., Che Y.Y., Li L., Zhu L.L., Chen G.S., Feng T. (2024). Phytogenic feed additives as Natural antibiotic alternatives in animal health and production: A review of the literature of the last decade. Anim. Nutr..

[6] Samolińska W., Grela E.R., Kowalczuk V.E., Kiczorowska B., Klebaniuk R., Hanczakowska E. (2019). Evaluation of garlic and dandelion supplementation on the growth performance, carcass traits, and fatty acid composition of growing-finishing pigs. Anim. Feed Sci. Technol..

[7] Hu G., Lu Y., Yu W., Ding Q., Yang Q., Zhou J., Ma Z. (2014). A steroidal saponin from the seeds of *Allium tuberosum*. Chem. Nat. Compd..

[8] Sharifi-Rad J., Mnayer D., Tabanelli G., Stojanović-Radić Z.Z., Sharifi-Rad M., Yousaf Z., Vallone L., Setzer W.N., Iriti M. (2016). Plants of the genus *Allium* as antibacterial agents: From tradition to pharmacy. Cell. Mol. Biol..

[9] Hong H., Niu K.M., Lee J.H., Cho S., Han S.G., Kim S.K. (2016). Characteristics of Chinese chives (*Allium tuberosum*) fermented by *Leuconostoc mesenteroides*. Appl. Biol. Chem..

[10] Zhang W.N., Zhang H.L., Lu C.Q., Luo J.P., Zha X.Q. (2016). A new kinetic model of ultrasound-assisted extraction of polysaccharides from Chinese chive. Food Chem..

[11] Kothari D., Lee W.D., Kim S.K. (2020). *Allium* flavonols: Health benefits, molecular targets, and bioavailability. Antioxidants.

[12] Lee W.D., Moon S.G., Lee A.R., Kim K.I., Kim J.I., Ga G.W., Kim Y.G., On J.Y., Jeon S.W., Kim S.H. (2025). Investigation of egg productivity, egg quality, egg storability, organ characteristics, gut microbiota, and health parameters with different addition levels of fermented *Allium tuberosum*. Braz. J. Poult. Sci..

[13] Xie Y., Kumar S.T., Zou H., Luo T.T., Zhang Y.P., Zhang Q., Li Y., Niu K.M., Zhai Z.Y., Wang C.F. (2025). Integrated transcriptomic and metabolomic analysis reveals regulatory effects of fermented Chinese chive on early testicular development in piglets. Antioxidants.

[14] Lee W.D., Kothari D., Moon S.G., Kim J., Kim K.I., Ga G.W., Kim Y.G., Kim S.K. (2022). Evaluation of non-fermented and fermented Chinese chive juice as an alternative to antibiotic growth promoters of broilers. Animals.

[15] Niu K.M., Kothari D., Lee W.D., Cho S., Wu X., Kim S.K. (2020). Optimization of Chinese chive juice as a functional feed additive. Appl. Sci..

[16] Kothari D., Lee W.D., Jung E.S., Niu K.M., Lee C.H., Kim S.K. (2020). Controlled fermentation using autochthonous *Lactobacillus plantarum* improves antimicrobial potential of Chinese chives against poultry pathogens. Antibiotics.

[17] AOAC (1990). Official Methods of Analysis.

[18] Babiker S.A., El Khider I.A., Shafie S.A. (1990). Chemical composition and quality attributes of goat meat and lamb. Meat Sci..

[19] Folch J., Lees M., Sloane-Stanley G.H. (1957). A simple method for the isolation and purification of total lipids from animal tissues. J. Biol. Chem..

[20] Zhang Y., Erdene K., Ao C., Bao Z., Du H., Feng Z., Umair A., Chen B. (2021). Effects of *Allium mongolicum* Regel and its extracts supplementation on the growth performance, carcass parameters and meat quality of sheep. Ital. J. Anim. Sci..

[21] Xie K., Wang Z., Wang Y., Wang C., Chang S., Zhang C., Zhu W., Hou F. (2020). Effects of *Allium mongolicum* Regel supplementation on the digestibility, methane production, and antioxidant capacity of Simmental calves in northwest China. Anim. Sci. J..

[22] Zhao Y., Erdene K., Bao Z., Ao C., Bai C. (2022). Effects of *Allium mongolicum* Regel essential oil supplementation on growth performance, nutrient digestibility, rumen fermentation, and bacterial communities in sheep. Front. Vet. Sci..

[23] Puvača N., Ljubojević D., Kostadinović L.J., Lukač D., Lević J., Popović S., Đuragić O. (2015). Spices and herbs in broilers nutrition: Effects of garlic (*Allium sativum* L.) on broiler chicken production. Worlds Poult. Sci. J..

[24] Lee S.H., Bang S., Jang H.H., Lee E.B., Kim B.S., Kim S.H., Kang S.H., Lee K.W., Kim D.W., Kim J.B. (2020). Effects of *Allium hookeri* on gut microbiome related to growth performance in young broiler chickens. PLoS ONE.

[25] Hai P.Y., Anh L.X., Hoa N.X. (2025). Fermented chive (*Allium schoenoprasum*) with *Lactobacillus plantarum*: A potential antibiotic alternative feed additive for broilers challenged with *Escherichia coli*. Fermentation.

[26] Son J., Lee W.D., Kim H.J., Kang B.S., Kang H.K. (2022). Effect of providing environmental enrichment into aviary house on the welfare of laying hens. Animals.

[27] Ao X., Yoo J.S., Zhou T.X., Wang J.P., Meng Q.W., Yan L., Cho I.H., Kim I.H. (2011). Effects of fermented garlic powder supplementation on growth performance, blood profiles and breast meat quality in broilers. Livest. Sci..

[28] Omer H.A., Ahmed S.M., Abdel-Magid S.S., El-Mallah G.M., Bakr A.A., Abdel Fattah M.M. (2019). Nutritional impact of inclusion of garlic (*Allium sativum*) and/or onion (*Allium cepa* L.) powder in laying hens’ diets on their performance, egg quality, and some blood constituents. Bull. Natl. Res. Cent..

[29] Wang Z.B., Ding L.N., Liu J.J., Savarin P., Wang X.L., Zhao K., Ding W.Y., Hou Y.L. (2023). Allicin ameliorates glucose and lipid metabolism via modulation of gut microbiota and bile acid profile in diabetic rats. J. Funct. Foods.

[30] Yang Z., Cui Z., Zhang M., Sun L., Zhao L., Su L., Jin Y. (2025). Analysis of the effects of hydroxyl radicals on the volatile flavor composition and lipid profile of sheep meat based on HS-SPME-GC-MS and UPLC-MS/MS studies. Food Chem..

[31] Vasta V., Ratel J., Engel E. (2007). Mass spectrometry analysis of volatile compounds in raw meat for the authentication of the feeding background of farm animals. J. Agric. Food Chem..

[32] Grabež V., Bjelanović M., Rohloff J., Martinović A., Berg P., Tomović V., Rogić B., Egelandsdal B. (2019). The relationship between volatile compounds, metabolites and sensory attributes: A case study using lamb and sheep meat. Small Rumin. Res..

[33] El Hadi M.A., Zhang F.J., Wu F.F., Zhou C.H., Tao J. (2013). Advances in fruit aroma volatile research. Molecules.

[34] Hauck T., Weckerle B., Schwab W. (2000). Metabolism of ethyl tiglate in apple fruits leads to the formation of small amounts of (R)-ethyl 2-methylbutanoate. Enantiomer.

[35] Roth M., Meiringer M., Kollmannsberger H., Zarnkow M., Jekle M., Becker T. (2014). Characterization of key aroma compounds in distiller’s grains from wheat as a basis for utilization in the food industry. J. Agric. Food Chem..

[36] Haag F., Hoffmann S., Krautwurst D. (2021). Key food furanones furaneol and sotolone specifically activate distinct odorant receptors. J. Agric. Food Chem..

[37] Wang N.B., Zhong Y.Y., Zhu H.Q., Zhao H.Y. (2026). Correlations between fatty acids and key aroma compounds in roasted beef cuts for flavor customization. Front. Nutr..

[38] Bleicher J., Ebner E.E., Bak K.H. (2022). Formation and analysis of volatile and odor compounds in meat—A review. Molecules.

[39] Chen J., Tan X., Weng Y., Zhao R., Che H., Irwin D.M., Zhang S., Li B. (2025). Comparative analysis of flavoromic and metabolomic profiling differences between red, firm and non-exudative (RFN) and pale, soft and exudative (PSE) pork. LWT.

[40] Zhu Y., Chen J., Chen X., Chen D., Deng S. (2020). Use of relative odor activity value (ROAV) to link aroma profiles to volatile compounds: Application to fresh and dried eel (*Muraenesox cinereus*). Int. J. Food Prop..

[41] Li X., Han B., Liu D., Wang S., Zhao J., Wang L., Li R., Bao G., Pei Q., Sun D. (2025). Transcriptomic and flavor metabolomic exploration of the genetic basis of meat quality and flavor in Tibetan sheep. BMC Genom..

[42] Zhang Y., Diao Y., Raza S.H.A., Huang J., Wang H., Tu W., Zhang J., Zhou J., Tan Y. (2025). Flavor characterization of pork cuts in Chalu black pigs using multi-omics analysis. Meat Sci..

[43] Piao M.Y., Lee H.J., Yong H.I., Baek S.H., Kim H.J., Jo C., Wiryawan K.G., Baik M. (2019). Comparison of reducing sugar content, sensory traits, and fatty acids and volatile compound profiles of the longissimus thoracis among Korean cattle, Holsteins, and Angus steers. Asian-Australas. J. Anim. Sci..

